# Perceived peer norms, health risk behaviors, and clustering of risk behaviors among Palestinian youth

**DOI:** 10.1371/journal.pone.0198435

**Published:** 2018-06-21

**Authors:** Peter Glick, Umaiyeh Khammash, Mohammed Shaheen, Ryan Brown, Prodyumna Goutam, Rita Karam, Sebastian Linnemayr, Salwa Massad

**Affiliations:** 1 Millennium Challenge Corporation, Washington, DC, United States of America; 2 Juzoor for Health and Social Development, Ramallah, Palestine; 3 Al Quds University, Abu-Dis, Palestine; 4 RAND Corporation, Santa Monica, California, United States of America; 5 Palestinian National Institute of Public Health, Ramallah, Palestine; Leibniz Institute for Prevention Research and Epidemiology BIPS, GERMANY

## Abstract

Relatively little is known about patterns of health risk behaviors among Middle Eastern youth, including how these behaviors are related to perceived peer norms. In a sample of approximately 2,500 15–24 year old Palestinian youth, perceived engagement of general peers in alcohol consumption, drug use and sexual activity was substantially greater than youths’ own (self-reported) engagement in these activities, suggesting a tendency to overestimate the prevalence of risk-taking behavior among peers. Individual participation in a risk behavior strongly covaries with the perceived levels of both friends’ and peers’ engagement in that behavior (p = 0.00 in each case). In addition, significant clustering of risk behaviors is found: youth who participate in one risk behavior are more likely to participate in others. These findings for a rare representative sample of Middle Eastern youth are strikingly similar to those in the US and Europe. The clustering of behaviors suggests that prevention programs should be structured to deal with a range of connected risk behaviors for which certain youth may be at risk. The findings also suggest that adjusting expectations about peers’ behavior may reduce young Palestinians’ engagement in risk taking.

## Introduction

Health risk behaviors among adolescents and youth are a global concern. Smoking, drug and alcohol use during adolescence have long been recognized as having direct health implications and may increase the risks of developing chronic dependence and illness in adulthood [1,2]. Research in the U.S. and other contexts has revealed several recurrent patterns in youth health risk behaviors. First, youth tend to perceive high (and may possibly overestimate) engagement of peers in risk behaviors relative to their own levels of engagement [3,4,5]. Second, their likelihood to engage in such behaviors themselves is positively related to these descriptive peer norms [6,7,8]. Third, youth who engage in one risk behavior tend to engage in others, that is, behaviors are clustered [9]. This pattern is often explained by problem behavior theory, introduced by Jessor [10,11], whereby an underlying behavioral syndrome causes a youth to adopt multiple risk behaviors.

These patterns have important policy implications. The correlation of an individual’s behavior with perceived peer behavior suggests that influencing what youth think about peers—or possibly, influencing peers’ actual behavior—may reduce their likelihood of engaging in risk activities. Clustering of risk behaviors would suggest that to be effective, prevention education programs need to deal with a range of connected behaviors for which certain youth may be at risk, not just single behaviors such as drug use.

Very little is known about these patterns of behavior among youth in the Middle East and among Palestinian youth in particular. Surveys in the region mostly use school-based convenience samples of adolescents rather than representative, random samples of youth that include out of school as well as older youth, who may be at greatest risk. They do not ask about many risk behaviors (in particular, sensitive ones such as sexual activity) or do so only in terms of perceptions regarding peers, not the youth’s own engagement in an activity; surveys gathering data both on own behavior as well as perceptions of peers are especially rare. Yet youth risk behavior, including alcohol and drug use and earlier initiation of sexual activity, is a rising concern in the region with implications for health and for HIV risk specifically [12,13,14].

Further, few studies, either in the Middle East or elsewhere, distinguish perceptions of the behaviors of close friends (proximate peers, or individuals in one’s social network), and of more general peers, that is, the broader cohort of individuals of the same sex and age in the community. Both may be important for determining an individual’s behavior. While it might be expected that the behavior of friends plays a particularly important role, perceptions of general peers may also be influential. Further, youth are more likely to be uncertain about—and hence potentially overestimate—risk behaviors of general peers, so that there may be more opportunity for interventions that correct these perceptions of norms.

The present paper uses a rare representative sample of Palestinian youth to investigate the relationship of individual behavior to the perceived behavior of peers, distinguishing close friends (proximate peers) and general peers. We examine the overall validity of general peer perceptions by considering the extent to which they are similar for youth within the same community. We also examine the covariance of individual risk behaviors, measured as the odds ratio of engaging in one health risk behavior conditional on engaging in another.

A few previous studies of young people in the region, using school-based samples, have addressed one of the patterns of concern to this study, the clustering of risk behaviors. In a study of younger adolescents age 11–15 living in Israel, the West Bank, and Gaza, engagement in multiple risk behaviors (defined broadly to include smoking, bullying, excess time with friends, parental disconnectedness, and several others) was substantially more likely than engaging in just one, among those who engaged in any risk behavior [15]. Multiple risk behaviors were more common among Israeli youth, both Jewish and Arab, than among Palestinians in the West Bank and Gaza. A study of Lebanese university students [16], using a sample closer in age to the one in the present study and a more similar set of risk behaviors, reports that nonmedical users of psychoactive prescription medications have higher likelihoods of also abusing alcohol and using illegal drugs. An earlier study of Lebanese university students [17] also found evidence of clustering of risk behaviors, defined to include (lack of) exercise, nutritional eating, and seatbelt use, in addition to alcohol use, sexual behavior, and fighting.

In the present study, we investigate the present of multiple health risk behaviors in a random, non school-based sample of Palestinian youth age 15–24 as well as examining the relationships of own behavior to perceptions of peers’ behavior. In addition to assessing whether patterns observed in primarily Western contexts are also found in a sample of Middle Eastern youth, the findings will also be of use for developing appropriate prevention programs, which remain limited in the Palestinian Territories as elsewhere in the region despite growing concerns over youth health risk activities.

This study builds on prior analysis of the data from the survey, known as the Palestinian Youth Health Risk Study [18,19], that shows generally low but not insignificant prevalence of most health risk behaviors among young people, including alcohol use, drug use, and sexual activity before marriage. For example, 22.4% of male youth aged 20–24 and 11.6% of females reported having tried alcohol (8.1% and 3.6% for male and female youth age 15–19). 9.3% of unmarried male youth and 6.7% of unmarried female youth age 20–24 report having had sexual intercourse, although almost one quarter of both report any sexual experience. Smoking (cigarettes or waterpipe), on the other hand, is strikingly high, even among younger youth (45.4% of males and 21.2% of females 15–19 smoke). Rates of interpersonal violence (fighting) are also significant, and appear higher than among similar aged youth in the US but comparable to rates in South Africa, another setting with a history of political and social conflict.

## Methods

### Participants and procedures

The survey targeted a representative sample of 2,500 youth 15–24 in the West Bank and East Jerusalem. A stratified two-stage random sample was drawn from the 2007 population census, with strata formed by crossing the 12 governorates with urban, rural, and refugee camp location. Within strata, 208 survey clusters (census enumeration areas) were randomly sampled with probability proportional to size. Within each cluster, 14 households with youth in the appropriate age range were selected using a modified random walk [19]. In cases where households had more than one individual age 15–24 of the targeted gender, Kish tables were used to randomly select the youth for interview. The survey interviewer (always of the same sex as the targeted youth) briefly interviewed the household head or a parent to get basic household demographic and other information, and then conducted the main youth interview. In some urban areas, it proved difficult to find households with youth. Therefore in some sample clusters a considerable number of residences had to be visited before households with youth were identified. If the selected youth was temporarily not home at the time of the initial visit, survey staff were instructed to make arrangements to return when the youth was at home, and at least two follow-ups attempts were made to interview the youth.

Youths’ oral consent/assent and (for minors) parents’ or guardians’ oral consent for interviews was obtained after interviewers explained the purpose and content of the survey. Oral rather than written consent was deemed appropriate to the survey content and environment, as participants could provide sensitive information without a written record that could potentially identify them. The study adhered to the guidelines of the Declaration of Helsinki. Ethical approval for the consent procedure and for all other study procedures was granted by RAND Corporation Human Subjects Protection Committee. Substantial efforts were made to develop procedures to ensure that youth were comfortable discussing sensitive topics. Interviewers were strictly instructed to ensure that the youth interview was conducted in a private room or other private area (e.g., the roof of the house). Youth could choose to be interviewed at a local youth center or other outside location, though few did so. To accommodate sensitivities, questions on sexual activity were not asked of minors (those under 18).

Interviews were conducted face-to-face, with interviewers reading questions aloud and writing down responses, with one partial exception. For questions on sexual activity, which were deemed to be the most sensitive, respondents were given the option of using a self-administered (paper) questionnaire (SAQ) for questions on sexual activity. Here the questions were still read aloud by the interviewer, but the answers were written by the youth and placed in a sealed envelope. After initial fieldwork revealed that very few youth chose the SAQ, possibly reflecting a lack of understanding of the method, it was decided to randomly allocate youth to SAQ or face to face for sexual activity questions to ascertain if the mode mattered for responses. This analysis will form the focus of a separate study.

Refusal rates by parents/household head or youth were almost uniformly low—11% for the survey overall. They were significantly higher (about 30%) in the area of East Jerusalem, an urban location formally annexed by Israel in 1980 and marked by significant social and political tensions as well as security concerns on the part of residents. Rates of non-response (“No answer” or “Don’t know”) on individual risk behavior questions were generally very low—under 1%. Rates were somewhat higher (though under 5%) for questions on current drug use, asked of those who indicated that they had tried drugs [19]. In the interview, after answering questions on a range of relatively non-sensitive topics, youth were asked their perceptions of risk behaviors (smoking, alcohol use, drug use, and violence or fighting) among general peers and proximate peers, and then asked about their own activities. This order was selected to avoid respondents shaping answers about peers to be consistent with what they had reported (and possibly, under-reported) about their own risk activity.

For the *general* peers’ questions, respondents were asked to think about youth in the community of their own age and sex, beginning with less potentially sensitive questions about the share of peers who are working and the share using tobacco before asking about the share engaging in more sensitive behaviors. The survey responses suggest that respondents were able to give meaningful answers in this format [19]. For example, there was little clumping of responses at 50%, indicating that youth took time to think about their perceptions of these shares instead of simply resorting to the modal response. For *proximate* peers, youth were asked about three individuals their own age and sex who were closest to them (individuals “who you spend your time with, such as your good friends”) and who we will refer to below as ‘friends’.

### Measures

#### Smoking

Youth were asked first if they had ever tried smoking tobacco (including both cigarettes and *narghila* or water pipe), and if so, if they currently smoked. (Unless otherwise noted, shares or percentages presented in this paper for current behavior are based on all youth in an age/gender group, not conditional on ever having engaged in the activity.)

#### Alcohol use

Youth were asked first if they had ever tried alcohol, and if so, if they currently drank on occasion.

#### Drug use

Separate ‘ever used’ questions were asked for marijuana or hashish, pills, inhalants, and cocaine or heroin. Youth who said they had ever tried any of these drugs were asked if they currently used any drugs.

#### Sexual activity

Youth were asked if they had ever had experience of sexual activity with a member of the opposite sex, defined as “romantic kissing, touching private body parts, or sexual intercourse”. The question was asked only of unmarried, non-minor (over 17 years) youth. Those reporting affirmatively were then asked specifically if they had ever had sexual intercourse (SI), defining the term explicitly to avoid ambiguity.

#### Violence

Respondents were asked if they had been involved in a physical fight with someone in the last year, and how many times.

#### Behavior of general peers

Youth were asked to estimate the share (percentage) of young people their age and sex in their community who engage in the following behaviors: smoking, current drinking, current drug use, and (among those not married) sexual intercourse.

#### Behaviors of proximate peers

For the three individuals of their own age and sex closest to them, youth were asked how many of the three engaged in smoking, current drinking, current drug use, and sexual intercourse if not married. For comparisons with own and general peers’ rates of engagement in the behavior, friends responses are expressed below as shares—that is, 0,1,2, or 3 out of 3 total.

### Data analysis

Analysis of differences in behavior by subgroups was done primarily using Pearson chi-square tests. To examine the covariance of individual risk behaviors, we used logistic models to estimate odds ratios of a youth engaging in one behavior conditional on engaging in another, with controls for age and location. Separate analyses were performed by gender. The analysis used STATA version 13, incorporating the two-stage survey design, in particular to allow correlations of standard errors within sample clusters.

## Results

### Peers’ and friends’ behaviors

Table 1 presents means for self-reported engagement in different behaviors as well as means of perceived engagement of proximate peers (close friends) and general peers. Prevalences of own behaviors by age, gender, and location are discussed in detail elsewhere [18]. Perceived risk activity engagement of friends follows the same patterns by gender, age and location as own behaviors: higher among older youth, among males, and in urban areas and refugee camps relative to rural areas. However, point estimates for mean friends’ engagement are usually moderately higher than means of self-reported own engagement. For example, for male youth 20–24, the mean own smoking prevalence is 71.5%, while it is 76% for friends (p = 0.030 for the difference); for young women in this age group the shares are 31% and 29% respectively (p = 0.018). For current alcohol use in this age group, 9% of males say they currently drink, compared with 13% for friends (p = 0.000); the corresponding figures for females are 4% and 6%. (p = 0.001).

**Table 1:** Own risk behaviors and perceptions of friends’ and peers’ behaviors (% engaging in activities).

		Males				Females			
		all	urban	rural	camps	All	urban	rural	camps
**Age**		**Current smoking**							
**15–19**	Self	45.44	46.53	41.92	48.28	21.55	26.79	9.95	20.41
	Friends	54.07	53.40	52.19	66.09	16.87	18.71	11.52	21.53
	Peers	63.99	63.72	60.28	79.18	20.08	23.19	10.60	28.52
**20–24**	Self	71.52	77.57	56.62	73.68	31.22	36.60	16.08	34.04
	Friends	76.38	76.98	72.06	86.84	27.61	32.71	13.05	31.21
	Peers	80.36	80.45	78.76	85.26	28.78	34.13	11.66	39.11
		**Current alcohol use**							
**15–19**	Self	3.35	4.49	0.51	3.45	1.19	1.85	0.00	0.00
	Friends	6.41	7.28	3.72	8.19	1.93	2.62	0.70	0.68
	Peers	13.00	13.37	10.86	17.27	5.54	7.36	1.11	7.12
**20–24**	Self	9.09	11.21	3.68	10.53	4.06	5.04	1.40	4.26
	Friends	13.02	15.26	7.16	14.91	6.71	8.22	2.33	7.97
	Peers	22.47	25.11	15.24	25.53	10.86	13.26	3.11	15.16
		**Current drug use**							
**15–19**	Self	1.21	1.43	1.01	0.00	0.15	0.23	0.00	0.00
	Friends	1.03	1.23	0.00	2.87	0.25	0.31	0.18	0.00
	Peers	7.60	8.53	3.78	12.71	4.19	5.65	0.55	6.32
**20–24**	Self	3.64	4.98	0.74	2.63	1.23	1.86	0.00	0.00
	Friends	3.98	5.10	1.23	4.39	2.12	2.57	0.70	2.90
	Peers	13.17	15.24	8.51	12.15	8.10	10.14	0.96	14.05
		**Sexual activity, unmarried (intercourse)**							
**15–19**	Self	5.49	6.92	1.72	5.00	4.06	4.48	1.96	8.33
	Friends	4.20	3.99	4.73	4.17	9.79	8.94	10.67	14.07
	Peers	7.68	7.74	7.03	9.31	10.64	12.38	5.32	17.30
**20–24**	Self	9.33	12.16	2.29	11.76	6.85	8.84	2.56	3.57
	Friends	11.80	13.58	7.45	13.06	20.81	22.18	16.11	25.98
	Peers	14.15	15.37	11.74	11.82	15.21	18.41	5.10	20.00

Notes: ‘Friends’ refer to three closest friends of the respondent. % for each respondent is calculated as the number reported to engage in the behavior divided by 3. ‘Peers’ refer to general peers in the community of the same age and sex as the respondent

In contrast, for general peers (youth of same age and gender in their community), respondents perceive prevalences of behaviors that are substantially higher than their own reported engagement in these behaviors and that of close friends. For example, whereas 9.1% of older male youth say they currently drink alcohol and the mean proportion of close friends reported to drink is 13%, the mean perceived rate of drinking among general age-sex peers is 22%; for females in this age group the rates are 4.1% and 6.7% for own and friends’ drinking, respectively, and 9% for peers. A similar pattern prevails for drinking among younger youth of both genders. For drug use, the difference between own and friends’ reported use (both of which are very low) on the one hand, and peers on the other, is even larger in proportional terms.

It may be noted that if youth were fairly well aware of how their peers behave, responses within a community about these peers should be relatively consistent, as the questions in effect ask all respondents in an age/sex category to estimate the same datum—the share of youth like themselves in the community who engage in a behavior. Therefore these responses, if they are capturing the local prevalence of a behavior, should be relatively highly correlated within sample clusters (of which there are 208 in the survey), and should also be more highly correlated than the intra-cluster responses for own engagement in the behavior, as these do truly vary across individuals within a community. The association of responses within a cluster can be measured with the intracluster correlation coefficient (ICC), the ratio of between-cluster variation over the sum of the total (within-cluster and between-cluster) variation; a higher ICC indicates stronger consistency or relatedness of an outcome within clusters.

ICCs for own and peer engagement are shown in Table 2. For own engagement in behaviors, which are binary outcomes, we use the approach of Rodriguez and Elo [20] to derive the ICCs and their confidence intervals. As shown, for males, ICCs for (own) smoking and drinking are low (.031 and .056); these are similar to school-based ICCs in studies of US students [21]. Consistency within clusters of responses regarding both oneself and one’s peers is stronger for female than male youth. However, a uniform finding for both genders is that ICCs for responses about local peer engagement in a behavior are substantially larger than for responses about the individual’s own behavior. That is, a larger share of the overall variation in responses about peers comes from differences across communities rather than differences within them, compared to the case of responses about own behaviors. Although it is not possible to state unambiguously what a ‘high’ value would be for ICCs (they cannot be interpreted as simple Pearson correlation coefficients), the relative consistency in responses about local peer behavior suggests that the peer prevalence responses are meaningful.

**Table 2:** Intracluster correlation coefficients (ICCs) for own and peers’ engagement in risk behaviors.

	Males		Females	
Behavior/Respondent engagement	Own engagement	Peer engagement	Own engagement	Peer engagement
Smoking	0.031	0.179	0.343	0.447
Alcohol use	0.056	0.269	0.127	0.575
Drug use	0.106	0.358	0.018	0.592
Ever Sexual intercourse (unmarried)	0.156	0.263	0.201	0.544

Notes: ICC is the ratio of between-cluster variation divided by the total variation, the sum of the within-cluster and between-cluster variation. For smoking, alcohol use, and sexual intercourse, ‘own engagement’ refers to current self-reported participation of the respondent and ‘peer engagement’ refers to the perceived share of local age/sex peers participating. For drug use, own engagement refers to the respondent reporting ever trying drugs and peer engagement refers to the perceived share of peers currently engaged in drug use. All peer and own behavior ICCs are significant at the 1% level.

### Correlation of self-reported own behaviors and that of friends and peers

Table 3 examines the relationship of the respondent’s own behavior to the perceived behaviors of friends and general peers. The table compares the mean of friends’ and peers’ prevalences for respondents who report engaging in a behavior with those who report not engaging in the behavior. In general, the differences are very large and statistically significant, with youth who report engaging in a given behavior also reporting higher friends’ as well as peers’ engagement in the behavior (p = 0.00 in each case). In proportional terms, the differences are largest for drug use. Among male youth who say they have never tried drugs, mean reported friends’ (current) usage is just 1% compared with 17% for those who say they have tried drugs (p = 0.000). Mean reported general peers’ drug use for male youth who have tried drugs is 8% compared with 26% for those who have not (p = 0.000). Patterns for females are very similar. Although in both cases the associations are strongly significant, the correlations appear larger between own and friends’ behaviors than between own and peers’ behavior.

**Table 3:** Means of friends’ and peers’ engagement in risk behaviors by respondent’s engagement in the behavior (%).

		Males				Females			
Behavior and respondent engagement		Friends	*p*	Peers	*p*	Friends	*p*	Peers	*p*
**Current smoking**	No	0.43	0.000	61.95	0.000	0.09	0.000	15.60	0.000
	Yes	0.79		77.37		0.57		47.54	
**Current alcohol use**	No	0.06	0.000	15.49	0.000	0.03	0.000	6.93	0.000
	Yes	0.59		38.67		0.45		46.55	
**Ever tried drugs**	No	0.01	0.000	8.36	0.000	0.01	0.001	5.30	0.000
	Yes	0.17		25.87		0.14		28.94	
**Ever Sexual intercourse (unmarried)**	No	0.06	0.000	10.93	0.000	0.19	0.000	11.68	0.000
	Yes	0.46		32.31		0.72		45.67	

Notes: For drugs and sexual intercourse, questions regarding friends and peers ask about their current engagement behavior, not whether they ever engaged in it. Reported p-values are from regressions of perceived friends or peers shares on the respondent’s own self- reported engagement in the behavior, with controls for age and location (urban, rural, refugee camp).

### Covariance of individual risk behaviors

Table 4 presents odds ratios of engaging in one health risk behavior conditional on engaging in another, based on logistic models for males and females with controls for age and location (urban, rural, refugee camp). For young men, the associations of individual risk behaviors are very large. For example, the odds that a male youth who is a tobacco smoker also currently consumes alcohol are about 9 times higher than for a male youth who does not smoke (p = 0.000); the odds of having ever used drugs are 3.8 times higher (p = 0.000); of having had sexual intercourse, about 11 times higher (p = 0.001). There is also an association of smoking and engaging in violence but this is somewhat lower: male youth who smoke are about 1.6 times more likely to have been in a physical fight in the last year (p = 0.001) and the associations of fighting with other risk behaviors are also generally smaller than between the other risk behaviors. However, all odds differences in the table for male youth are significant at p<0.05.

**Table 4:** Associations of individual risk behaviors (odds ratios).

**Males 15–24**					
	**Current smoking**	**Current alcohol use**	**Ever used drugs**	**Ever had sexual intercourse**	**Ever had sexual activity**
**Current smoking**	--	9.486	3.843	10.988	3.916
*P*	--	0.000	0.000	0.001	0.000
**Current alcohol use**	9.486	--	9.453	19.974	9.145
*P*	0.000	--	0.000	0.000	0.000
**Ever used drugs**	3.843	9.453	--	11.031	8.249
*P*	0.000	0.000	--	0.000	0.000
**Ever had sexual intercourse**	10.988	19.974	11.031	--	--
*P*	0.001	0.000	0.000		
**Ever had sexual activity**	3.916	9.145	8.249	--	--
*P*	0.000	0.000	0.000		
**Females 15–24**					
	**Current smoking**	**Current alcohol use**	**Ever used drugs**	**Ever had sexual intercourse**	**Ever had sexual activity**
**Current smoking**	--	--	8.017	22.499	3.984
*P*			0.000	0.000	0.000
**Current alcohol use**	--	--	3.646	3.394	2.670
*P*	--	--	0.062	0.075	0.041
**Ever used drugs**	8.017	3.646	--	6.839	2.115
*P*	0.000	0.062	--	0.001	0.081
**Ever had sexual intercourse**	22.499	3.394	6.839	--	--
*P*	0.000	0.075	0.001		
**Ever had sexual activity**	3.984	2.670	2.115	--	--
*P*	0.000	0.041	0.081		

Notes: Based on logit regressions. Shows the increase in the likelihood of engaging in an activity (shown in first column) if the individual engages in another activity (along top row). Model also includes controls for age and location (urban, rural, camp). Among females, all who reported current drinking also reported currently smoking so this relationship is not estimated

For female youth, the correlations are similarly positive but more variable and less precisely estimated. All of the (small number of) female youth reporting alcohol use also smoke, so no odds ratios are estimated for alcohol use conditional on smoking. The relationship of other behaviors to smoking is very strong for females, with ORs of 8.01 for ever tried drugs (p = 0.000), 3.98 for any sexual activity (p = 0.000) and 4.23 for fighting (p = 0.000). Alcohol use appears to be significantly associated with a higher likelihood of sexual activity and internet/phone sex.

To test whether covariances of behaviors changes as youth get older, the same models were run adding interactions of age with the given behavior indicator. With the exception of smoking and sexual intercourse for females, for which the age interaction was positive, interaction results were not statistically significant.

## Discussion

This study is among the first in the Middle East region to collect representative information on health risk behaviors of youth, and is further distinguished by the fact that information was collected on perceived behaviors of both close friends and general peers. With respect to several key patterns, the findings from this sample of Palestinian youth display a striking similarity to youth or adolescent surveys carried out in other regions, even if overall levels of engagement in risk behaviors appear lower than elsewhere other than for smoking. These patterns include (1) perceived peer norms for risk behaviors that are substantially higher than self-reported engagement in these behaviors; (2) a correlation of a youth’s own behavior with these perceived peer norms; and (3) a strong likelihood that youth who engage in one risk behavior also engage in others.

Regarding (1), it should first be noted that the disparities in own and perceived peers’ risk behaviors are fairly small for close friends but large for general age-sex peers in the community. This suggests that youth overestimate engagement in risks among individuals whose behavior they do not closely observe. This is not surprising to find in our sample given that large disparities between descriptive peer norms and self-reported alcohol and drug use behavior have been noted for years in the literature in the US and elsewhere [3,5,8]—though confirmation of this pattern in a Middle East context has been lacking.

However, youth may also be underreporting their own—and close friends’—risk behavior in this conservative environment. Some evidence from US studies of adolescent or youth drug use, using biomarkers among other means, suggest underreporting [22]. Therefore the extent to which the discrepancy in own and peer rates of risk behavior reflects overestimation of the latter or understating of the former remains unclear, though as we discussed elsewhere [18] it is likely that actual prevalences of behaviors in this population fall between the means for individual self-reports and peers. Finally, it should be emphasized that even if youth overestimate prevalence among general peers, these perceptions are not random guesses. The high intracluster correlations of these responses reported above indicate consistency among youth in the same cluster with respect to perceptions of general peer behavior.

Pattern (2), the strongly positive correlation of individuals’ self-reported risk behavior with perceived descriptive peer norms—whether of friends or general peers—is also consistent with studies noted above from outside the region. Like those studies, our results suggest a possible causal link from perceptions of peer engagement to an individual’s own participation in health risk activities. Here the emphasis is less on whether these perceptions are accurate than whether they influence one’s own behavior. However, as is well recognized, correlations of self-reported behavior and perceived peer behavior may reflect selection issues or confounders rather than a causal relation. Those participating in a stigmatized activity may simply have better information about how common that activity is in the community; they may tend to associate with, hence know more about, peers who are most like themselves in terms of behavior (homophily); they may assume that other youth are like themselves in terms of behavior (projection); or, finally, youth who engage in a behavior may tend to exaggerate the extent of that behavior among others as a means of self-justification. Each of these factors can explain the correlation of own behavior and perceived peer engagement, apart from any causal relationship. It is difficult to arrive at a conclusive determination on causality, especially with cross-section data; this is even the case with studies collecting longitudinal data with direct information on friends’ behavior [23].

Finally, as in research in other settings [9], we find that youth who participated in one risk behavior have an elevated chance of participating in other risk behaviors. This pattern is often explained by problem behavior theory [10,11], whereby an underlying behavioral syndrome causes a youth to participate in multiple risk behaviors. We find that ‘traditional’ health risk behaviors such as smoking and drinking are linked not only to each other but also to engagement in interpersonal violence (fighting), as has been observed in surveys of adolescents in Western countries [24]. Some research from industrialized countries that examines changes in clustering of risk behaviors as young people transition to adulthood find that the correlations decline with age, suggesting a weakening of an underlying problem behavior syndrome, though other studies find no change [9]. In our sample, we find few differences in the correlations of behaviors between younger and older youth. Future analysis of the data will examine whether youth who participate in multiple risk behaviors share important characteristics, namely a lack of protective factors such as family support and income or an excess of potential risk factors such as exposure to violence or depression.

## Limitations and conclusions

An important limitation of this study, discussed in the preceding section, is the reliance on respondents’ self-reports of risk behaviors, which may lead to underreporting. Still, the strong relationships among measures reported here are expected to be robust in a qualitative sense and are consistent with those found in more thoroughly researched environments. They have potentially important implications for policies to prevent or reduce risk behaviors among Palestinian and other Middle Eastern youth. Since the patterns are like those observed in other regions, in principal, program design can take similar approaches as in the U.S. and elsewhere, with appropriate testing and adaptation for local cultural context.

For example, the correlation of an individual’s behavior with perceived peer behavior suggests that influencing what youth think about peers may reduce their likelihood of engaging in risk behaviors [25]. In particular, the possibility that youth overestimate the extent of what peers are doing implies that provision of more accurate information on peers would be useful. This assumes that the relationship of these perceptions and an individual’s own behavior is causal. Given the considerations noted above, this cannot be inferred from our results. However, a substantial evaluation literature examines the efficacy of interventions to alter youth perceptions of peer norms and consequently, their risk behaviors; many of these programs focus on drinking among university students in US settings. Such interventions appear successful in changing perceptions about peers and many also are able to change actual behaviors, though overall, success in the latter regard is mixed [25,26,27]. In contexts where such ‘feedback’ interventions have been successful, both an association of own and perceived peer risk taking and an apparent overestimation of peer risk behavior engagement are observed, as in the present sample. The literature suggests (with some exceptions) that youth behavior is more responsive to information about norms of those who are most like them in terms of factors such as gender and interests [26]; in the present context, it is possible that information needs to reference more specific groups than our measure of general same-sex peers in the community. Careful design and testing are needed to see if such interventions can be successful in the Palestinian context and the region generally. This would be a worthwhile focus of future research, given the dearth of interventions addressing substance use for Middle Eastern youth.

With regard to the covariance of individual risk behaviors, it is clear that, as in other contexts, prevention education programs for Palestinian youth should be structured to deal with a range of connected risk behaviors for which certain youth may be at risk, not just single behaviors such as drug use [2]. Again, careful design and testing is important, as there are few extant examples from the region of such programming.

## Supporting information

S1 Data (DTA)

S2 Data Documentation. (PDF)
