# Supplementary material for: Perceived peer norms, health risk behaviors, and clustering of risk behaviors among Palestinian youth
Source: PLoS One. 2018 Jun 21;13(6):e0198435. doi: 10.1371/journal.pone.0198435 (PMC6013164; doi:10.1371/journal.pone.0198435)
Supplement: S2 Data — (PDF) [file pone.0198435.s002.pdf]

VARIABLE DESCRIPTION

variable	type	label	variable label	description/comments
cluster_newid	float			Sample cluster randomly renumbered
m1	byte	sex1	Gender	
m14	byte	m14	Current marital status	
prb1	int	prb1	What percent of male youth your age are employed	Asked about youth of the same age and sex in the community (Wording for male respondent; gender reversed for females)
prb2	int	prb2	What percent of male youth are tobacco smokers	" "
prb3	int	prb3	What percent of male youth take alcohol	" "
prb4	int	prb4	What percnet of male youth use drugs	" "
prb5	int	prb5	What percent of unmarried male youth have sexual relations	" "
prb6	byte	prb6	Three people closest: how many are married	Asked about the three people closest to respondent, of same age and sex.
prb7	byte	prb7	Three people closest: how many are smokers	" "
prb8	byte	prb8	Three people closest: how many take alcohol	" "
prb9	byte	prb9	Three people closest: how many use drugs	" "
rb1	byte	rb1	Do you use tobacco (e.g. cigarettes, shisha/Goza)	Currently use tobacco
rb8	byte	yesno	Taken an alcoholic drink such as beer,wine	Ever had alcohol
rb10	byte	yesno	Do you currently take alcoholic drinks	Asked if ever had alcohol
rb19	byte	yesno	Have you every tried marijuana or hashish	
rb21	byte	yesno	Have you tried amphetamine or trip pills	
rb23	byte	yesno	Have you tried inhaled or smelled substances as drugs	
rb25	byte	yesno	Have you tried cocaine or heroine	
rb29	byte	yesno	These days, do you take drugs	Asked if ever used any of above drugs
rb36	byte	yesno	Have you every had any sexual activity with a female	"Have you ever had any sexual activity with a female? By this I mean romantic kissing, touching private body parts, or sexual intercourse". (For male respondent; gender reversed for females)
rb37	byte	yesno	Have you ever had sexual intercourse with a female	Asked if ever had any sexual activity (For male respondent; gender reversed for females)
areatype	byte	area1	type of area urban=1, rural=2, camp=3	
urban	byte		areatype==Urban	
rural	byte		areatype==Rural	
refugee_camp	byte		areatype==Camp	
age18plus	float			
age_group3	float	age_grou	p3	
age_groupALL	float	age_grou	p3ALL	
current_smoke	float			Variables for analysis created from 'rb' variables above, with No Answer and Don't Know responses (998,999) converted to missing.
ever_drink	float			" "
current_drink	float			" "
ever_drugs	float			" "
ever_married	float			" "
ever_sex_activity	~y float			" "
ever_intercourse	~e float			" "
female	float			
male	float			
unmarriedabove18	18 float			
age15_19	float			
age20_24	float			

DESCRIPTIVE STATISTICS

Variable	Obs	Mean	Std. Dev.	Min	Max
cluster_ne~d	2481	104.364	60.0488	1	208
m1	2481	1.499798	.5001008	1	2
m14	2481	1.399839	2.790057	1	96
prb1	2481	47.51229	126.1629	0	999
prb2	2444	47.5671	35.71155	0	100
prb3	2330	12.41159	20.33441	0	100
prb4	2269	7.917585	16.33929	0	100
prb5	2168	11.57841	19.22443	0	100
prb6	2481	.5530028	.9116805	0	3
prb7	2477	1.2717	1.235276	0	3
prb8	2476	.197496	.5557503	0	3
prb9	2475	.049697	.2688994	0	3
rb1	2478	3.069411	2.997648	1	98
rb8	2478	2.010896	3.356656	1	98
rb10	258	1.98062	6.021008	1	98
rb19	2477	2.012111	1.936141	1	98
rb21	2469	1.99352	.0802557	1	2
rb23	2469	1.980559	.1380972	1	2
rb25	2468	1.994327	.075118	1	2
rb29	104	4.432692	16.21085	1	98
rb36	1207	2.164043	6.198052	1	98
rb37	291	1.694158	.4615568	1	2
areatype	2481	1.424023	.6317948	1	3
urban	2481	.6533656	.475994	0	1
rural	2481	.2692463	.4436576	0	1
refugee_camp	2481	.0773881	.2672602	0	1
age18plus	2481	.6146715	.4867709	0	1
age_group3	2481	2.708182	1.612542	1	6
age_group3~L	2481	1.428053	.4948963	1	2
current_sm~e	2476	.4099354	.4919208	0	1
ever_drink	2475	.1054545	.3072003	0	1
current_dr~k	2468	.0409238	.1981539	0	1
ever_drugs	2463	.046691	.2110191	0	1
ever_married	2479	.124647	.3303849	0	1
ever_sex_a~y	1202	.234609	.4239306	0	1
ever_inter~e	1200	.0733333	.2607915	0	1
female	2481	.4997985	.5001008	0	1
male	2481	.5002015	.5001008	0	1
unmarried~18	2481	.4901249	.5000033	0	1
age15_19	2481	.5719468	.4948963	0	1
age20_24	2481	.4280532	.4948963	0	1
